# Metropolitan Hospitals Ancient and Modern

**Published:** 1889-01-12

**Authors:** 


*(To be continued.)*

### Metropolitan Hospitals Ancient and Modern.

(By a Medical Superintendent.)

*(Continued from page 215.)*

### III.—THE FRENCH SYSTEM—MEDICAL SCHOOLS.

We are not in the habit of imitating the French in many of their undertakings, less perhaps in the organisation of charity than in anything else, but anyone interested in hospital work might do worse than examine for himself the constitution of the "Association de l'Assistance Publique" at Paris, which is partly a municipal but also a charitable organisation. This body has the supervision of the hospitals, hospices, and other charitable foundations, issuing its orders and supplies from a central office in the heart of Paris, and publishes annually a full report with statistical tables showing its income and expenditure in minute detail. From a recent "Compte Morale" of the administration it would seem that the cost per bed in each of the Parisian hospitals is greatly in excess of what it was twenty years ago, but it is far from having arrived at the high standard of the London hospitals. In the account referred to, the average cost of an occupied bed in the general hospitals amounted to £50 14*s.* 2*d.*, in the special hospitals to £55 8*s.* 4*d.*, and in the children's hospitals to £40 6*s.* 3*d.* It may be as well to note that the French hospitals are not encumbered with out-patients. The want of a similar supervision in London hospitals is not only the cause of great waste of public money, but it precludes any possibility of discovering in which hospital or department of the hospital our haphazard system is defective, whereas a good standard of comparison and accounts, framed on a similar basis would prove advantageous in every way. The Council of the Hospital Sunday Fund has succeeded so far, in obtaining certain special returns from the medical charities applying for grants, which might be still further developed by the Council insisting on the authorities of the separate hospitals adopting one uniform plan in the drawing up of their balance-sheets and setting out their expenditure in full detail, not in block only, but for patients and households respectively.

Apart from the relief given to the sick and maimed the fact that hospitals are the only means by which young men entering the medical profession can receive practical instruction is usually advanced as a strong argument for their support; but while it cannot be disregarded, it is doubtful whether an educational purpose of this kind addresses itself to the multitude in as cogent a form as the direct relief of suffering. The original founders of the hospitals may not have had any conception that the buildings they were rearing would afterwards become important schools of medicine and surgery, but as time went on it was found absolutely necessary in populous centres throughout the country that they should become so. In university towns the local hospitalsh ave always been more or less affiliated with the university in which the main medical teaching was carried on; but in London, from the want of any such facilities, medical education in all its branches had to be taught within the walls of the hospitals, and mainly by the medical officers attached to them. It is clear that without this voluntary help, it would have been hopeless to have attempted any national scheme of medical education; nevertheless, in complying with the enactments of the medical corporations and examining boards, the hospitals incurred responsibilities, and encountered monetary difficulties from which institutions established by the State for curative purposes are entirely exempt. In foreign countries the duty of making this provision falls either on the government or municipal authorities, and if it is difficult to understand how a generous legislature would sanction the arrangement without also contributing to its efficiency, it is harder still to believe that the scanty revenues of hospitals and of most charitable institutions should be called upon to reimburse the exchequer many thousands annually in London alone, to relieve the very class which the hospitals are doing their very best to prevent becoming a burden on the rates. There is every reason to believe that this anomalous impost, which is of comparatively recent date, will be repealed as soon as it is brought under the notice of Parliament.

If it be thought by some that the appanage of a medical school involves the hospital in additional expenditure, and interferes somewhat with its harmonious working, it must be remembered that the extra cost of maintaining, and frequently of originating buildings in connexion therewith, even in the case of the hospitals best able to bear the strain, falls on the income derived from students' fees, while the advantages accruing from an alliance which secures the elite of the medical profession to attend the sick, must be accounted no mean gain. It is also a matter of grave importance to the two thousand or more students carrying on their studies in London that they should have thoroughly competent instructors, and it would be a cause for lasting regret if, by any unfortunate combination of circumstances, the field for clinical instruction should be in any way abridged. To enlarge this field it has often been suggested that the large infirmaries and sick asylums should be utilised, as the diseases under treatment in these establishments, with the exception of accidents, are similar in most respects to those admitted to the general hospitals. This may be the case, but there are difficulties in the way of combining the two services almost insurmountable. Do what we will, there will continue to exist a certain antagonism between legal relief and voluntary almsgiving, and the good people who encourage the sentiment are legion. The views and practices of the two systems are in frequent collision, and poor-law guardians make no secret of their aversion to co-operating with charitable trusts, whether of an individual or corporate character. Still, something might be done to relieve the periodical embarrassments of hospitals caused by lack of funds, which is illustrated by the fact that, while nearly a fourth of their certified accommodation remains empty, the vacant beds in the poor-law infirmaries do not constitute more than an eighth or a tenth part of their capacity. Instead of adding to or building new infirmaries, there is no reason why the local demand for accommodation could not be equitably adjusted between the parish authorities and the hospitals, the former undertaking to reimburse the hospitals for expenses incurred on a scale previously agreed upon, which would probably be about the same as the patients would cost in the union infirmary. Already overtures of this nature have been made to the hospitals by the managers of the sick asylums with respect to the admission of cholera and enteric fever in the event of epidemics, and have been met in a friendly spirit; but it would be manifestly more satisfactory and be more readily acceded to if the application referred to the ordinary class of non-infectious ailments. In the interests of both science and humanity, it is greatly to be regretted that such a system of mutual help has not been further developed, as it would enable the pauper sick to participate in those privileges which are so freely bestowed on those above them in the social scale. It would also enable the poor-law medical officer to send patients to the hospital for consultative advice and treatment, whose complaints may savour of doubt or difficulty, or who may require special surgical operations. It only requires some tact and forbearance on the part of the representatives of the two services to render such a system as is here sketched out practically workable.

